# *Nna1* gene deficiency triggers Purkinje neuron death by tubulin hyperglutamylation and ER dysfunction

**DOI:** 10.1172/jci.insight.136078

**Published:** 2020-10-02

**Authors:** Jianxue Li, Evan Y. Snyder, Fenny H.F. Tang, Renata Pasqualini, Wadih Arap, Richard L. Sidman

**Affiliations:** 1 Department of Neurology, Beth Israel Deaconess Medical Center, Harvard Medical School, Boston, Massachusetts, USA.; 2 Sanford Burnham Prebys Medical Discovery Institute, La Jolla, California, USA.; 3 Rutgers Cancer Institute of New Jersey and Division of Cancer Biology, Department of Radiation Oncology, Rutgers New Jersey Medical School, Newark, New Jersey, USA.; 4 Rutgers Cancer Institute of New Jersey and Division of Hematology/Oncology, Department of Medicine, Rutgers New Jersey Medical School, Newark, New Jersey, USA.

**Keywords:** Neuroscience, Neurodegeneration

## Abstract

Posttranslational glutamylation/deglutamylation balance in tubulins influences dendritic maturation and neuronal survival of cerebellar Purkinje neurons (PNs). PNs and some additional neuronal types degenerate in several spontaneous, independently occurring Purkinje cell degeneration (*pcd*) mice featuring mutant neuronal nuclear protein induced by axotomy (*Nna1*), a deglutamylase gene. This defective deglutamylase allows glutamylases to form hyperglutamylated tubulins. In *pcd*, all PNs die during postnatal “adolescence.” Neurons in some additional brain regions also die, mostly later than PNs. We show in laser capture microdissected single PNs, in cerebellar granule cell neuronal clusters, and in dissected hippocampus and substantia nigra that deglutamase mRNA and protein were virtually absent before *pcd* PNs degenerated, whereas glutaminase mRNA and protein remained normal. Hyperglutamylated microtubules and dimeric tubulins accumulated in *pcd* PNs and were involved in *pcd* PN death by glutamylase/deglutamylase imbalance. Importantly, treatment with a microtubule depolymerizer corrected the glutamylation/deglutamylation ratio, increasing PN survival. Further, before onset of neuronal death, *pcd* PNs displayed prominent basal polylisosomal masses rich in ER. We propose a “seesaw” metamorphic model summarizing mutant *Nna1*-induced tubulin hyperglutamylation, the *pcd’s* PN phenotype, and report that the neuronal disorder involved ER stress, unfolded protein response, and protein synthesis inhibition preceding PN death by apoptosis/necroptosis.

## Abstract 2

Purkinje cell degeneration is due to ER stress, unfolded protein response, and protein synthesis inhibition preceding Purkinje neuron death by apoptosis/necroptosis.

## Graphical abstract

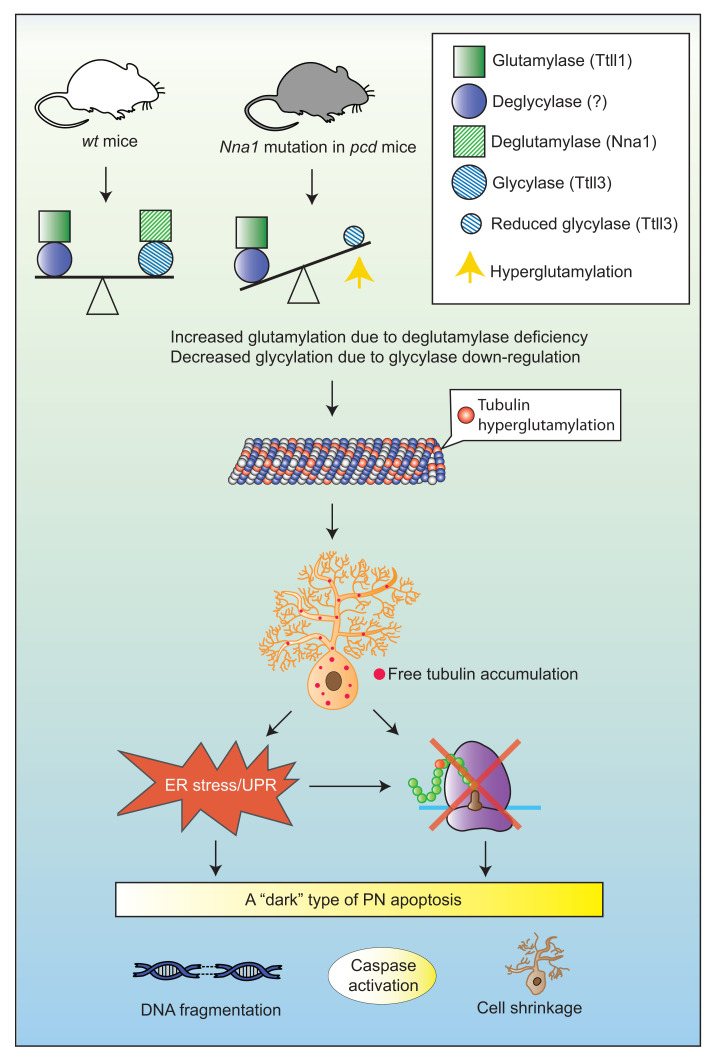

## Introduction

Tubulins are present in all eukaryotic animal and plant organisms (those with intracellular nuclei) from living single-cell organisms to humans, with free dimeric α- and β-tubulins plus microtubules, both types being crucial multifunctional cytoplasmic constituents. Glutamylation and glycylation are 2 of the most common genetic and enzymatic types of posttranslational modification of tubulin proteins (1–4). Each tubulin monomer possesses a C-terminal tail on its external surface, and the lengths and numbers of these tails per microtubule vary markedly, so that sequence identity among different tails is not readily established. Glutamylases bind postnatally to tails of both α- and β-tubulins. These glutamylases can be long lived or short lived, according to what additional peptides also bind.

In this study we evaluated the working hypothesis that tubulin polyglutamylation participates in the neuronal degeneration occurring in Purkinje cell degeneration (*pcd*) mutant mice (5, 6). Glutamylase and deglutamylase, respectively, add and remove polyglutamyl side chains of variable length on or from the exposed γ-carboxyl receptor groups of microtubule subsets of tubulin (5, 7, 8). Glutamylation, initially discovered on tubulins, regulates microtubule stabilization and several interactions between microtubules and their partners such as tau, microtubule-associated proteins, and microtubule-based molecular motors. In the brain, tubulin tyrosine ligase–like 1 (Ttll1) is a major glutamylase that catalyzes microtubulin and free dimeric (α and β) tubulin polyglutamylation, whereas neuronal nuclear protein induced by axotomy (Nna1), also cited by many authors as cytosolic carboxypeptidase-1, is a major deglutamylase that shortens polyglutamated chains of tubulins (5). Furthermore, it is inferred that free α- and β-tubulin glutamylation and deglutamylation play functional roles in several additional neuronal and microtubule dynamic events (9) as well as other key functions, including normal and pathological cell division and migration.

Notably, in addition to glutamylation and deglutamylation, another tubulin-modifying agent, a glycine ligase (glycylase) termed Ttll3 opposes Ttll1-induced tubulin glutamylation. Indeed, tubulin glycylation competitively opposes tubulin glutamylation, thereby also participating in regulation of microtubule polymerization and stabilization (4, 10).

The *Nna1* gene was discovered as an upregulated gene in mouse spinal cord neuronal cell bodies in response to their axons having been cut peripherally (11) and, subsequently, surprisingly, identified as the mutated gene, *pcd*, a spontaneously inherited mouse disease (12). Homozygous *pcd* mice feature abnormal maturation of Purkinje neuron (PN) dendrites beginning on about P10 (13) and death of almost all PNs on P22–P35 (14), resulting in severe ataxia, and degeneration of a few additional neuronal types degenerating simultaneously with PNs or after the PN degeneration, such as a subgroup of auditory thalamic neurons degenerating between P50 and P60 (15) and a slower progressive degeneration of olfactory bulb mitral neurons (14, 16) and retinal photoreceptor neurons (14, 17, 18). Strikingly, *Nna1* was identified as a member of the cytosolic carboxypeptidase family (19, 20)*,* and its genetic or experimentally induced nonfunction resulted in a marked increase in polyglutamates, putatively constituting a direct basis for the PN degeneration (5). However, molecular pathways through which *Nna1* dysfunction impairs neuronal survival have not yet been elucidated. Tantalizingly, at least 9 different mutations of the *Nna1* gene have been found in mice, and the mouse *Nna1* gene is 87% homologous with the corresponding human *NNA1* gene, making it plausible that *NNA1* gene mutations may be involved also in certain human neurodegenerative diseases, as cited in Discussion.

We hypothesize that *Ttll1* and *Nna1* genes encode a pair of mutually counterbalancing enzymes that either add or remove glutamine side chains of proteins and thereby control neuronal survival by regulating free tubulin and microtubulin composition and attached protein composition. We have here uncovered a pathological pathway in PNs through which *dysfunction* triggers both accumulation of hyperglutamylated tubulins and decreased tubulin glycylation. These mutant mice also show neuronal features of ER stress (21), protein synthesis inhibition, cytoplasmic atrophy (22), and a dark type of cell death by apoptosis (23) or perhaps in combination with the more recently described necroptosis (24).

## Results

### Characterization of glutamylase, deglutamylase, and glycylase in PNs.

With an integrated portfolio of molecular and cellular techniques, including laser capture microdissection (LCM) global gene expression in single quantitative PCR (qPCR), IHC, and immunoblots, we have systematically examined major members of the glutamylation and glycylation families in LCM-isolated single PNs, in enriched PN cultures, and in intact isolated cerebellar cortices. Ttll1 is a major glutamylase, whereas normal Nna1 protein is the first known deglutamylase. Ttll3 is a typical glycylase, but no deglycylases have as yet been identified in eukaryotic cells.

**Figure 1:**
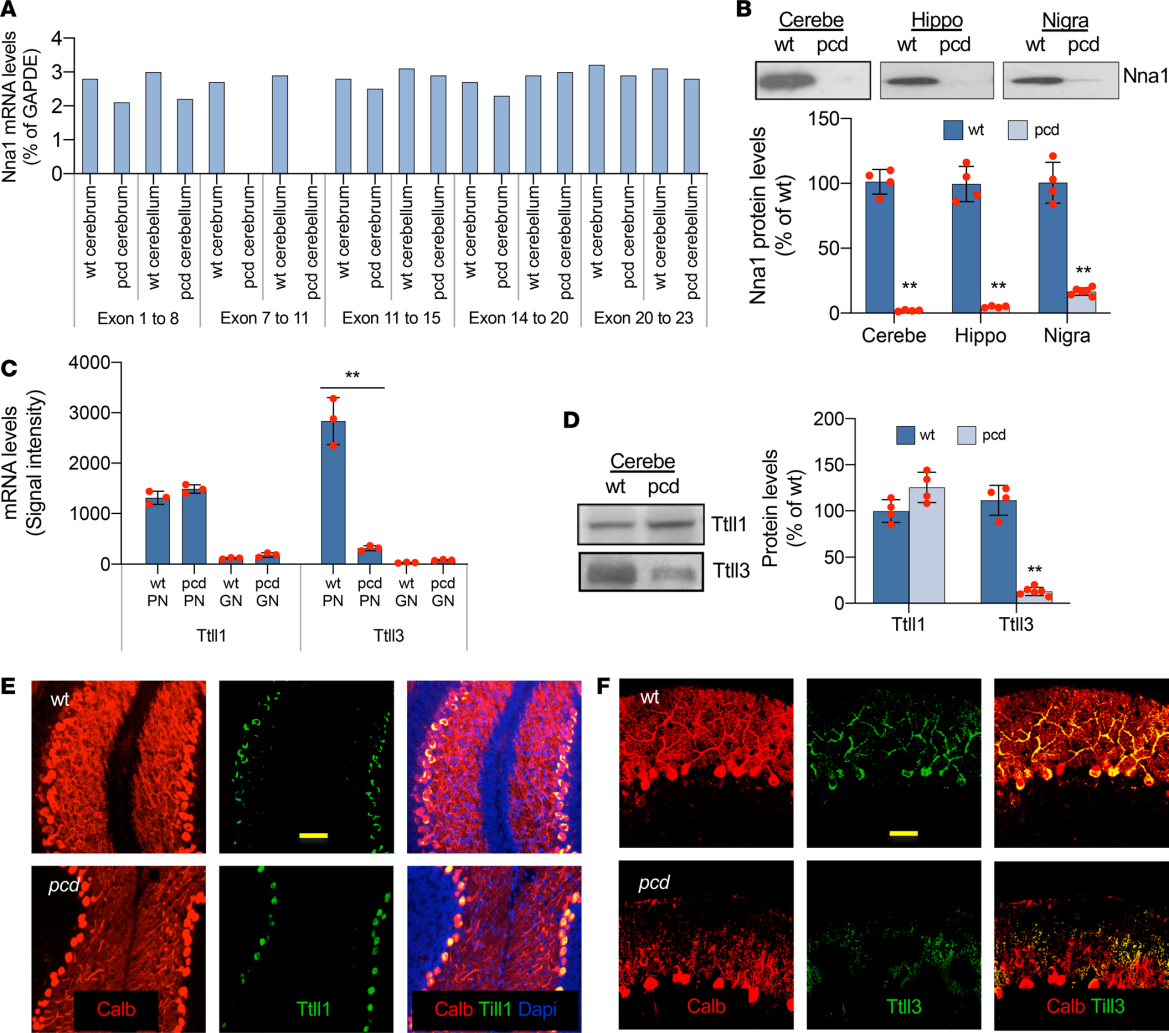
Characterization of Nna1, Ttll1, and Ttll3. (**A**) Nna1 mRNA lacks exons 7–11 in young adult *pcd^Sid^* cerebrum and cerebellum. (**B**) Nna1 protein is deficient in young adult *pcd* cerebellum, hippocampus, and substantia nigra. Dot pots with mean ± SD, *n* = 4–6; 2-tailed Student’s *t* test, compared with WT, **P < 0.01. (**C**) *Ttll1* and *Ttll3* mRNA were much higher in P20 WT PNs than in WT GNs. The *Nna1* mutation reduced *Ttll3* mRNA in P20 *pcd*. Dot pots with mean ± SD, *n* = 3; 2-tailed Student’s *t* test, compared with WT, **P < 0.01. (**D**) Ttll1 and Ttll3 blots were from 2 different gels. Ttll3 protein was decreased in P20 pcd cerebellum. Dot pots with mean ± SD, *n* = 4–6; 2-tailed Student’s *t* test, compared with WT, **P < 0.01. (**E**) Ttll1 protein (green) was equivalent in P20 WT and *pcd* cerebellum. Calbindin (Calb, red) in PNs, DAPI (blue) in cell nuclei. Scale bar: 50 μm. (**F**) Ttll3 protein (green) was decreased in P20 *pcd* PNs, like Calb (red) in the distal area of P20 *pcd* PN dendritic trees. Scale bar: 30 μm. Nna1, neuronal nuclear protein induced by axotomy; Ttll1, tubulin tyrosine ligase–like 1; *pcd*, Purkinje cell degeneration; PNs, Purkinje neurons; GNs, granule cell neurons.

Full-length *Nna1* mRNA in mouse cerebrum or cerebellum was divided into fragments (*n* = 5) in 5 pairs of primer sets. Its fragments 1–5 contain exons 1–8, 7–11, 11–15, 14–20, and 20–23, respectively. In *pcd^Sid^* mutant mice, fragment 2 has been eliminated due to an exon 7 deletion (13), but levels of fragments 1 and 3–5 were similar in WT and *pcd^Sid^* cerebra and cerebella (Figure 1A). Intriguingly, we found that the exon 7–deleted *Nna1* mRNA was not translated to normal Nna1 protein in P20 *pcd* cerebellum, hippocampus, and substantia nigra (Figure 1B), resulting in a major, and possibly even complete, brain deficiency of this crucial deglutamylase.

*Ttll1* mRNA levels were manyfold higher in PNs than in clusters of the very small and closely packed cerebellar granule cell neurons (GNs) of both P20 WT and *pcd* mice (Figure 1C), indicating the importance of protein glutamylation in cerebellar PNs. We did not recognize obvious changes in *Ttll1* mRNA and protein in P20 *pcd* cerebella (Figure 1D) or PNs (Figure 1, C and E) compared with WT controls. Furthermore, the *Ttll3* mRNA level was 20-fold higher in P20 WT PNs than in GNs (Figure 1C), which suggests a potential importance of protein glycylation in normal developing cerebellar PNs. Interestingly, the *Nna1* mutation markedly inhibited *Ttll3* mRNA expression (Figure 1C), leading to a negligible protein level of this glycylase in P20 *pcd* PNs (Figure 1, D and F). Because polyglutamylation appears to have a key role in posttranslational tubulin modification, we reasoned that the deficient deglutamylase and negligible glycylase induced by the *Nna1* gene mutation may have led to the heavy accumulation of hyperglutamylated free tubulins and microtubules in *pcd* PNs.

### Nna1 deficiency induced tubulin hyperglutamylation.

**Figure 2:**
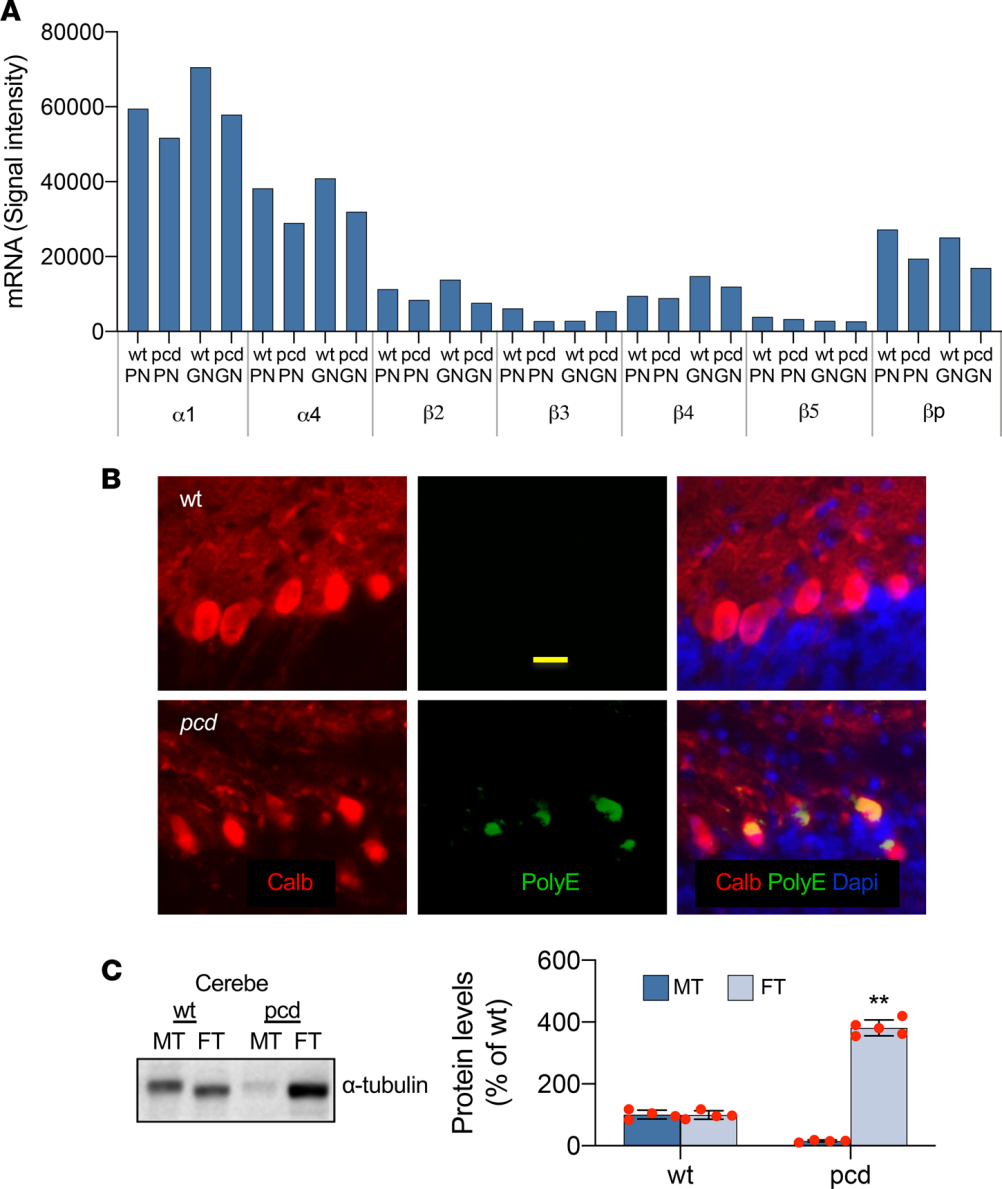
Tubulin subunits and microtubules. (**A**) mRNAs of major tubulin family members increased in P20 *pcd* PNs and GNs. (**B**) Hyperglutamylated tubulin (green) was found in P20 *pcd* PNs. Calb (red) in PNs, DAPI (blue) in cell nuclei. Scale bar: 15 μm. (**C**) Free dimeric tubulin proteins (FT) were strongly accumulated, whereas microtubules (MT) were nearly absent in P20 *pcd* cerebellum. Dot pots with mean ± SD, *n* = 4–6; 2-tailed Student’s *t* test, compared with WT, **P < 0.01. *pcd*, Purkinje cell degeneration; PNs, Purkinje neurons; GNs, granule cell neurons.

To test our working hypothesis, we further checked tubulin subunits and microtubules in LCM-isolated single PNs and grouped GNs as well as in intact cerebellar cortices. Microtubules are composed of tubulin subunits and form major cytoskeletal constituents oriented longitudinally in neuronal processes. Tubulin glutamylation is generally considered to contribute substantially to microtubule stability, whereas tubulin hyperglutamylation suppresses microtubule polymerization (25). Based upon our data on mRNA expression of the tubulin family (Figure 2A), we identified α1-, α4-, β2-, and β4-tubulin subunits as the predominant building blocks of microtubules in cerebellar PNs and GNs, among which α1-tubulin represents the most abundant subunit.

We next quantified protein levels of free dimeric tubulins, polyglutamylated tubulins, and microtubules by preparation of Triton X-100–soluble and –insoluble cytosolic fractions (26, 27). We found a greatly increased level of polyglutamylated tubulins in *pcd* cerebellar PNs (Figure 2B), data indicating that *Nna1* deficiency induced tubulin hyperglutamylation. We further explored the consequence of tubulin hyperglutamylation and recognized that the ratio of free dimeric tubulins versus microtubules was much higher in *pcd* than in WT cerebella (Figure 2C). These findings suggest an extensive accumulation of hyperglutamylated free dimeric tubulins in *pcd* mutant PNs, findings consistent with our working hypothesis of a “molecular seesaw.”

### Tubulin hyperglutamylation is a major factor responsible for pcd PN death.

**Figure 3:**
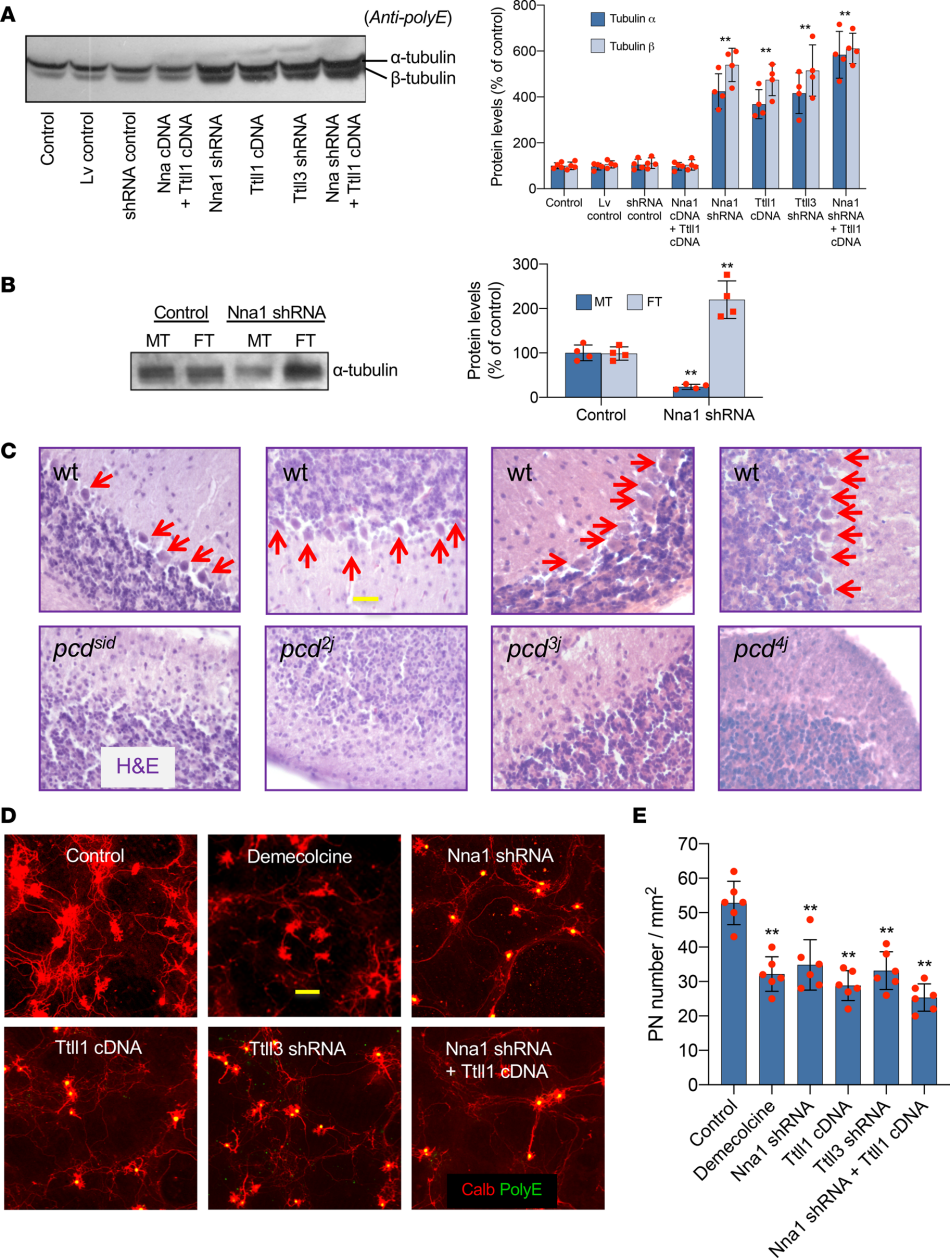
Tubulin hyperglutamylation and PN death. (**A**) Glutamylated tubulins increased in PN cultures given Nna1 shRNA (decreased deglutamylase), *Ttll1* cDNA (increased glutamylase), *Ttll3* shRNA (decreased glycylase), or combined *Nna1* shRNA plus *Ttll1* cDNA. Dot pots with mean ± SD, *n* = 4–6; 2-tailed Student’s *t* test, compared with control, **P < 0.01. (**B**) Free dimeric tubulin proteins (FT) were accumulated, whereas microtubules (MT) were decreased in Nna1 shRNA PN cultures. Dot pots with mean ± SD, *n* = 4–6; 2-tailed Student’s *t* test, compared with control, **P < 0.01. (**C**) H&E cerebellar cortices show total loss of PNs (arrows) in various alleles of P40 *pcd* mutants. Scale bar: 30 μm. (**D** and **E**) Images and quantification show decreases in PN survival in WT cerebellar cell cultures given demecolcine (microtubule depolymerizer), Nna1 shRNA, *Ttll1* cDNA, *Ttll3* shRNA, or combined *Nna1* shRNA plus *Ttll1* cDNA showed Calb (red) PNs PolyE-stained (green) tubulin hyperglutamylation. Dot pots with mean ± SD, *n* = 6; 1-way ANOVA, experimental (Nna1 shDNA, Ttll1 cDNA, Ttll3 shRNA, and Nna1 shRNA plus Ttll1 cDNA) groups compared with control group, **P < 0.01; no statistical difference among experimental groups or among control, Lv control, shRNA control, and Nna1 cDNA plus Ttll1 cDNA groups. Scale bar: 50 μm. PN, Purkinje neuron; Nna1, neuronal nuclear protein induced by axotomy; Ttll1, tubulin tyrosine ligase–like 1.

Microtubules constitute crucial cytoskeletal stabilizing constituents in WT PNs, whereas adult *pcd* mice feature loss of almost all PNs (14). We next explored whether posttranslational modifications in tubulin substructure are associated with *pcd* PN death. Based on our experimental data on tubulin hyperglutamylation in *pcd* in vivo, we further found in WT PN shRNA cultures that treatment with *Nna1* shRNA (*Nna1* knockdown), *Ttll1* cDNA (overexpression), *Ttll3* shRNA (Ttll3 knockdown), or combined *Nna1* shRNA plus *Ttll1* cDNA increased hyperglutamylated tubulin (Figure 3A), whereas cotreatment with WT *Nna1* (overexpression) plus *Ttll1* cDNA reduced the glutamylated tubulin level to normal (Figure 3A). Also, *Nna1* shRNA clearly increased the ratio of free dimeric tubulins to microtubules in PN cultures (Figure 3B).

Various mutant alleles of *pcd* in mice (*pcd^2J-4J^* and *pcd^Sid^*) share the same pattern and schedule of PN death (Figure 3C), as well as similar motor incoordination (13, 22). Thus, to verify the relationship between *pcd* PN tubulin hyperglutamylation and PN death, we next tested whether an accumulation of hyperglutamylated free tubulins would cause PN death in tissue cultures of WT PNs, GNs, or cerebellar mixed cells. We found that upon treatment with demecolcine (a microtubule depolymerizer), *Nna1* shRNA, *Ttll1* cDNA, *Ttll3* shRNA, or combined *Nna1* shRNA plus *Ttll1* cDNA suppressed PN survival (Figure 3, D and E). These results indicated that abnormalities in deglutamylase, glutamylase, and glycylase levels can influence PN survival in vitro, consistent with findings that *pcd* PNs were protected by experimental *Nna1* restoration (19, 28) and *Ttll1* knockdown (5) or deficiency (25).

### Free tubulin accumulation triggered ER stress in PNs.

**Figure 4:**
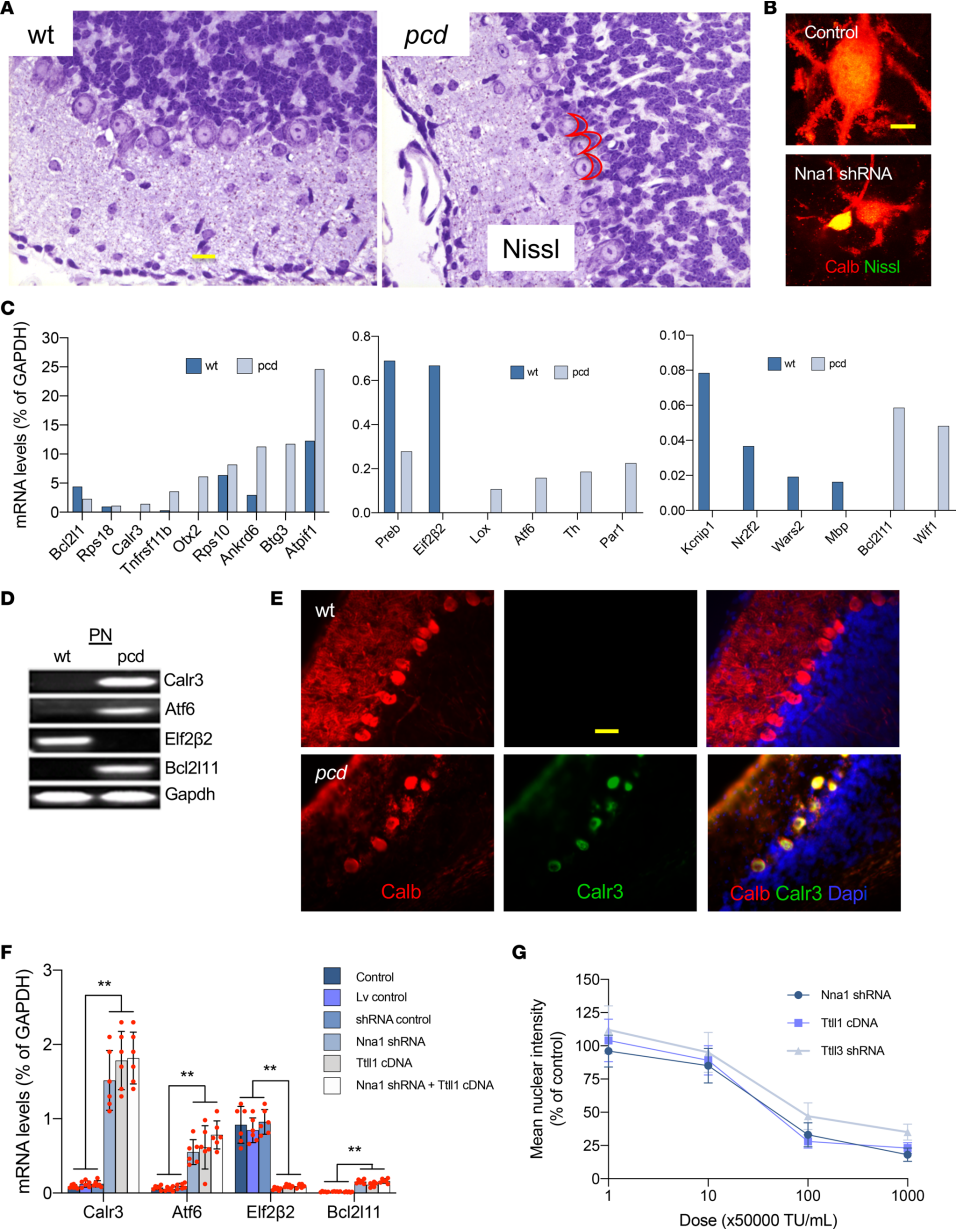
ER stress and protein synthesis inhibition. (**A**) Images of Nissl-stained cerebellar cortices show cytoplasmic basal polyribosome mass (inside red meniscus in the right panel) in P15 *pcd*. Scale bar: 15 μm. (**B**) *Nna1* shRNA induced abnormal polyribosomes (yellow), based on superimposed Nissl staining (green) and Calb (red) in cultured PNs. Scale bar: 15 μm. (**C**) Major changes of several mRNAs in P20 *pcd* PNs. (**D**) Increased ER stress markers (Calr3, Atf6, and Bcl2L11, an apoptotic initiator), and decreased protein synthesis initiator (Elf2β2) in P20 *pcd* PNs. (**E**) Cerebellar cortex images confirm the large increase of Calr3 (green) in P20 *pcd* PNs. Calb (red) PNs, DAPI (blue) cell nuclei. Scale bar: 15 μm. (**F**) Several markers show increased Calr3, Atf6, and Bcl2L11, plus decreased Elf2β2 in PN cultures given Nna1 shRNA (decreased deglutamylase), *Ttll1* cDNA, or combined *Nna1* shRNA plus *Ttll1* cDNA (increased glutamylase). Dot pots with mean ± SD, *n* = 6; 1-way ANOVA, experimental (Nna1 shDNA, Ttll1 cDNA, and Nna1 shRNA plus Ttll1 cDNA) groups compared with control group, **P < 0.01; no statistical difference among experimental groups or among control, Lv control, and shRNA control groups. (**G**) Protein synthesis curves show concentration-dependent decreases in PN cultures given *Nna1* shRNA, Ttll1 cDNA, or *Ttll3* shRNA (decreased glycylase shown as percentage of controls). There were no significant differences among *Nna1* shRNA, *Ttll1* cDNA, and *Ttll3* shRNA treatments. *pcd*, Purkinje cell degeneration; Nna1, neuronal nuclear protein induced by axotomy; PNs, Purkinje neurons; Calr3, calreticulin 3; Aft6, activating transcription factor 6; Bcl2L11, Elf2β2, eukaryotic initiator 2β2; B cell lymphoma 2–like protein 11; Ttll1, tubulin tyrosine ligase–like 1.

We next explored the pathological pathway(s) underlying hyperglutamylated free tubulin accumulation–mediated PN death. Cytoplasmic ER is the core organelle for protein synthesis, which participates in nascent folding, posttranslational modification, and transport to different intracellular locations (29). We identified that more than 30% of P15 (before onset of cell degeneration) *pcd* PNs have a prominent basal polyribosome mass (Figure 4A), composed of clustered ribosomes with abnormal ER and protein accumulation, as initially described by Landis and Mullen (21). Moreover, we found that the intense accumulation of hyperglutamylated free tubulins due to *Nna1* knockdown also produced abnormal polyribosomes in cultured WT PNs (Figure 4B).

Either disturbances in ER function or loss of ER integrity may result in ER stress, which in turn activates a series of signaling pathways, including the unfolded protein response (UPR) (30, 31). UPR counteracts original stress by changing expression of ER chaperones and regulators (32, 33). We found obvious increases in several ER molecules, such as calreticulin 3 (Calr3) and activating transcription factor 6 (Aft6), in P20 *pcd* WT controls (Figure 4, C–E), when the major phase of PN degeneration is just beginning. In addition, UPR counteracts original stress also by inhibiting protein synthesis, resulting in a decreased metabolic load within the ER (34). Protein synthesis machinery in the cytoplasmic rough ER is precisely assembled with polyribosome-associated nucleic acids and initiators (31). In fact, we detected a decrease of the eukaryotic initiator 2β2 (Elf2β2) by P20 in *pcd* cerebellar PNs (Figure 4, C and D).

To verify and confirm the adverse effects of hyperglutamylated free tubulins on ER regulators and polyribosome-associated initiators, we found that treatment with *Nna1* shRNA and/or *Ttll1* cDNA increased Calr3 and Aft6, and decreased Elf2β2 in cultured PNs (Figure 4F). *Nna1* shRNA, *Ttll1* cDNA, or *Ttll3* shRNA inhibited protein synthesis in PN cultures in a concentration-dependent manner (Figure 4G), a result indicative of specificity. Taken together, these findings strongly suggest that *Nna1* deficiency not only triggers ER stress and UPR but also inhibits protein synthesis via the high accumulation of hyperglutamylated free tubulins in *pcd* PNs in vivo and in PN cultures.

### ER impairment–mediated “dark” type of PN apoptosis.

**Figure 5:**
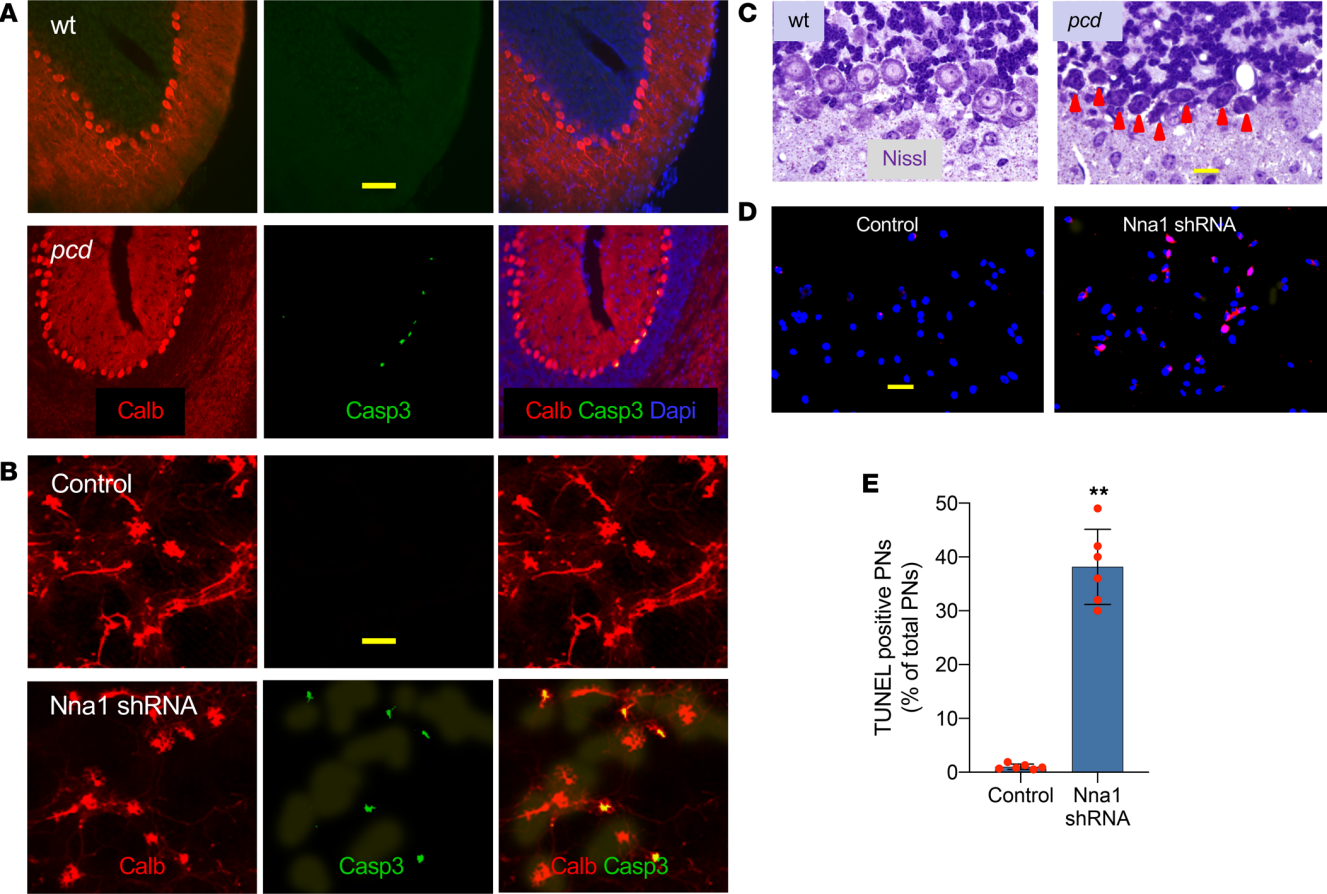
Dark type of PN apoptosis. (**A**) Cerebellar cortices show caspase-3 (Casp3) activation (green) in P20 *pcd* PNs. Calb (red) PNs, DAPI (blue) cell nuclei. Scale bar: 50 μm. (**B**) Cerebellar cell cultures show that *Nna1* shRNA moderately suppresses deglutamylation and induces Casp3 (green) activation in cultured PNs. Scale bar: 75 μm. (**C**) Cerebellar cortices show dark apoptotic PNs (arrowheads), nuclear condensation, and cellular shrinkage in P20 *pcd*s. Scale bar: 20 μm. (**D** and **E**) Increased PN TUNEL stain (red) in cell cultures given *Nna1* shRNA and DAPI (blue) cell nuclei. Dot plots with mean ± SD, *n* = 6; 2-tailed Student’s *t* test, compared with control, **P < 0.01. Scale bar: 50 μm. PNs*,* Purkinje neurons; *pcd,* Purkinje cell degeneration; Nna1, neuronal nuclear protein induced by axotomy.

When ER function is severely impaired, genes and pathways leading to cell death become activated (35). Caspase enzyme activation participating in protein substrate cleavage and subsequent cell disassembly is involved in ER stress–mediated apoptosis (36). We found a 10-fold increase in B cell lymphoma 2–like protein 11 (Bcl2L11, an apoptotic initiator) (Figure 4, C, and D) and Casp3 (Figure 5A) in P20 *pcd* PNs, followed by cell death of approximately 50% of *pcd* PNs by P23 and their nearly complete loss by P35. Tubulin hyperglutamylation also triggered Bcl2L11 increase (Figure 4F) and Casp3 activation (Figure 5B) in cultured PNs.

Cell shrinkage, DNA strand breaks, chromatin condensation, nuclear envelope degradation, nuclear blebbing formation, and fragmentation may occur at the late stage of apoptosis (23, 37, 38). A dark type of cell death represents an especially distinctive form of neuronal apoptosis that was mentioned in AMPA-induced PN death (39). We identified approximately 8% of PNs becoming darkly stained and shrunken in P20 *pcd* cerebellum (Figure 5C), data consistent with previous reports (38, 40). Also, we found DNA fragmentation and strand breaks in PN cultures that were repaired with *Nna1* shRNA (Figure 5, D and E). These findings indicate that *Nna1* deficiency triggers cerebellar PN apoptosis and possibly necroptosis (24, 41–43) via accumulation of hyperglutamylated free tubulins and ER impairment.

## Discussion

**Figure 6:**
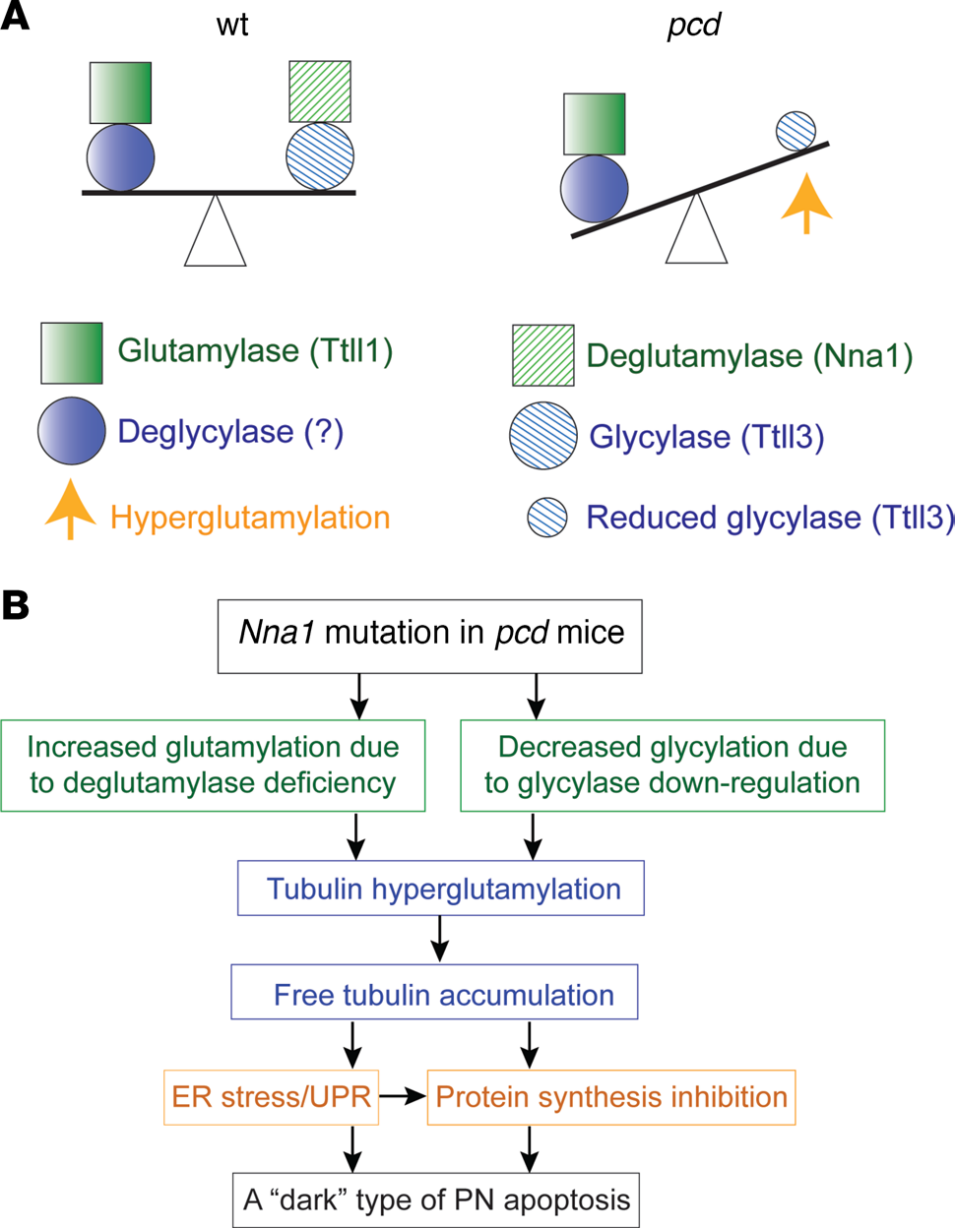
Schematic representation of the molecular mechanisms. (**A**) The molecular seesaw model on the left shows the WT balance between glutamylase (Ttll1) and deglutamylase (*Nna1*) on the seesaw’s left side and between glycylase protein (Ttll3) and a presumed deglycylase or other molecular mechanism on the seesaw’s right side. The molecular seesaw model on the right shows the *pcd* abnormal unbalance between the increased glycylase (measured in response to the net increase in tubulin that contains polyglutamate) and the decreased glycylation contrasting with the tubulin hyperglutamylation in *pcd* PNs, beginning at about P20. (**B**) The pathological pathway through which the *Nna1* mutation leads to a dark type of P20 *pcd* PN death. Ttll1, tubulin tyrosine ligase–like 1; Nna1, neuronal nuclear protein induced by axotomy; PNs, Purkinje neurons; *pcd,* Purkinje cell degeneration.

Our Discussion has been set toward a molecular seesaw model for tubulin posttranslational modification. Both sides of our seesaw models would be essentially in balance when 2 particular genes are functioning normally: the *Ttll1* gene, which adds glutamyl residues to selected α- and β-tubulin subunits, and the normal *Nna1* gene, which *de*glutamylates many of those glutamate residues (Figure 6A, left side). However, the seesaw model postulates (Figure 6B) that when there is a mutation in *Nna1* that eliminates this gene’s normal deglutamylating activity, as in *pcd* homozygous mutant mice, the side of the seesaw with the now-excessive glutamyls lies abnormally low (Figure 6A, right side). This seesaw is made further unbalanced by the *Ttll3* gene’s reduced activity, decreasing glycine residues almost to zero in mouse *pcd* mutants, although one might have expected cyclic protein side chains with glycines to have increased by binding in excess to the increased glutamates in *pcd* PN tubulins, so as to stabilize the glutamates. Yet we had checked the levels of tubulin glutamylase (*Ttll1*), tubulin deglutamylase (*Nna1*), and tubulin glycylase (*Ttll3*), as well as their roles in tubulin glutamylation and deglutamylation in WT and *pcd* cerebellar PNs, and found that no tubulin decyclase had yet been identified in any eukaryote cells, vertebrate, or otherwise. To date, only 2 metallopeptidases with decyclase activity are currently known, both of which are in the protozoan parasite *Giardia duodenalis* (44). Nonetheless, *Ttll3* is known to glycylate the β-tubulin tail at 4 sites in a hierarchical order. *Ttll3* competes with others of the several glutamylases, at least*Ttll7* and likely also *Ttll1*, for binding sites on tubulin tails, thus providing a partial molecular basis for the reversed correlation between glutamylation and glycylation. This opposite behavioral correlation demonstrates how 2 different posttranslational modifications can arise through the activities of related but distinct tubulin tyrosine ligase–like (TTLL) enzymes. To elucidate what structural elements differentiate *Ttll3* glycylases from glutamylases, both of which share the common TTLL scaffolds, further investigation is being defined by other investigators with the use of highly specific new Abs and crystallography.

In addition, we have uncovered a PN death pathway (Figure 6B) in which the *Nna1* functional deficiency due to any of several mutations in the *Nna1* gene in *pcd* mice not only resulted in microtubulin hyperglutamylation with similar effects on free tubulins but also stimulated ER stress, UPR, and protein synthesis inhibition. Severely impaired ER functions led further toward PN death. In both *pcd* PNs in vivo and in PNs in vitro, *Nna1* deficiency induced ER dysfunction and triggered dark PN apoptosis, cell shrinkage, caspase activation, and DNA fragmentation.

*Ttll3* glycylates β-tubulin tails and interacts there with the glutamylases *Ttll7* and likely *Ttll1* in molecular competition for binding to overlapping sites on tubulin tails, thus providing a molecular basis for an opposing correlation between increasing glutamylation and decreasing glycylation. Studies are underway to elucidate what structural elements differentiate glycylases from glutamylases, both of which share the common TTLL scaffold (e.g., 45, 46).

In sum, we have characterized glutamylase, deglutamylase, and glycylase in pathological mechanisms leading to PN death in dissected *pcd* cerebellum. Strong net glutamylation due to greatly decreased deglutamylation and glycylation induced accumulation of hyperglutamylated free tubulins in *pcd* PNs (Figure 1, Figure 2, and Figure 3) (4, 10), which in turn triggered ER stress, UPR, protein synthesis inhibition, and PN death (Figure 3, Figure 4, and Figure 5).

Glutamylation, a posttranslational modification, was first discovered with respect to α- and β-tubulins in the mid 1990s (1, 47). More recently, it was recognized that several deglutamylating enzyme proteins, apart from tubulins, also affect the behaviors of various additional cell types and functions (48). In tubulin molecules, glutamylation occurs at their carboxy-terminal tails, which, upon coordinated lengthwise assembly of microtubules, become exposed on the closely packed outer surfaces of the tubules, where they provide binding sites for several associated proteins and molecular motors. Mice deficient of glutamylase *Ttll1* display abnormal targeting of KIF1A and some key modulated synaptic functions (49). Normal deglutamylase (*Nna1*) is also involved in tubulin modification (5), and mutational loss-of-function of *Nna1* is an early deficit associated with PN degeneration in young *pcd* mice (12). Therefore, maintaining a functionally correct level of tubulin glutamylation by the coordinated actions of glutamylase, deglutamylase, and glycylase is essential. Accordingly, we here have put forward a hypothetical molecular seesaw model (Figure 6A), which potentially accounts for the dynamic balance among glutamylation, deglutamylation, and glycylation. If validated, this mechanistic model would have immediate biological value for the functional study of genetic mouse models and medical value for potential translational applications against currently intractable human neurodegenerative and perhaps malignant diseases.

Several other aspects of this work deserve more discussion. First, we uncovered that the *Nna1* mutation in *pcd* mice not only induced deglutamylase deficiency but also downregulated glycylase expression, leading to an accumulation of hyperglutamylated free tubulins in young postnatal PNs. These findings were also reproduced in cultured WT PNs given glutamylase overexpression (*Ttll1* cDNA) and deglutamylase or glycylase knockdown (*Nna1* or *Ttll3* shRNA). In contrast, the class of cerebellar cortical GNs — probably the most numerous neurons in the whole nervous system — contain little glutamylase (*Ttll1*) and glycylase (*Ttll3*). These negligible enzyme levels are perhaps factors in GN survival, contrasting with PNs in *pcd* mutant mice. The molecular mechanisms governing the slower, incomplete degeneration in *pcd* mice of olfactory bulb mitral neurons and retinal photoreceptor cells, as well as the acute severe degeneration of selected (auditory relay) thalamic neurons (but sharply limited to the later mouse age span of P50–P60), remain to be fully investigated. It also remains an open question whether the many other surviving classes of neurons in *pcd* mutants behave like cerebellar GNs or are otherwise spared.

Second, to elucidate how accumulated free tubulins mediate *pcd* phenotypes, we analyzed ER stress, UPR, and protein synthesis in young postnatal *pcd* PNs. The ER can recognize misfolded proteins without causing functional disruption, but when a more global disruption of protein folding overwhelms its coping mechanisms, UPR is activated. This cellular stress response related to ER will be activated in response to an accumulation of unfolded or misfolded proteins in the ER lumen. UPR may restore normal cell function by halting protein translation, degrading misfolded proteins, and activating signaling pathways, which in turn leads to increased production of ER molecules such as Calr3 and Aft6. Increased Calr3 binds mutated or misfolded proteins, leading them into a degradation path (50), whereas cleavage of Atf6 forms a transcription factor causing UPR (51). During protein synthesis, various initiation factors and regulators are produced. For instance, ElF2β2 is essential for protein synthesis by forming a tertiary complex with GTP and the initiator Met-rRNA (52), whereas decreased ElF2β2 in young *pcd* PNs suggests that deficiency of the first known deglutamylase, *Nna1*, may impair protein synthesis. If ER stress is prolonged and/or intensive, and if UPR fails to restore normal cell function within a limited time span, apoptosis is almost certain to be triggered. Cellular signals involved in ER stress–mediated apoptosis are complex, generally including activated caspases and additional factors that participate in protein cleavage and subsequent cell disassembly (36). Increased in young *pcd* PNs is apoptotic initiator Bcl2L11, a “death ligand” that neutralizes certain members of the prosurvival Bcl2 subfamily (53). Caspase3 is activated by abnormalities in ER stress and UPR pathways in young *pcd* PNs becoming apoptotic (54, 55). The late apoptotic phase is characterized by cell shrinkage, chromatin condensation, and DNA fragmentation. Young PNs in *pcd* cerebellum become darkly stained and shrunken, a dark type of cell apoptosis that has also been reported in AMPA-induced PN death (39) and *pcd^Sid^*; and which probably represents an especially distinctive form of neuronal degeneration. First, our studies in vivo and in vitro confirm that deficiency of deglutamylase *Nna1* may result in PN apoptosis via free tubulin accumulation–induced abnormal ER stress and UPR (Figure 6B). Second, another potentially relevant factor recently suggested (56) proposes that a nucleolar stress response may participate in PN degeneration in *pcd* mice.

Third, sustained overactivation of UPR has been implicated in many severe human neurodegenerative diseases, including Creutzfeldt-Jakob, Parkinson’s, and Huntington’s diseases, thus inhibiting UPR, and could become useful for disease therapy (57). Because the mouse *Nna1* gene is vulnerable to mutation, with 9 spontaneous mutant alleles on record (*pcd^1J-8J^* and *pcd^Sid^*) and the corresponding human gene *NNA1* (NM_015239) is 87% homologous with mouse *Nna1* (NM_023328), one might speculate that loss of *NNAl1* function may also regulate pathophysiological processes in certain human neurodegenerative and other types of disease, particularly those showing ER stress, UPR, and microtubule destabilization. For example, Alzheimer’s disease pathology involves misfolded Aβ in plaques and hyperphosphorylated tau in tangles. Either mutated amyloid precursor protein inducing ER stress/UPR (58–60) or tau hyperphosphorylation–mediated microtubule destabilization (61–63) might perhaps be a factor underlying neuronal death in this major disease. Therefore, it would seem pertinent to investigate *Nna1* deglutamylase roles in selected human neurodegenerative diseases. In fact, even in nondegenerative conditions such as human malignant diseases, international studies have already been initiated on targeting molecular features that may involve similar microtubule-based mechanisms governing cell cycle phases, interactions with dividing cells, cell migration to metastatic sites, and even platelet release from the bone marrow microenvironment into the peripheral blood circulation (64–66).

Fourth, one must acknowledge the strong experimental research program on tubulin biology and pathology under the leadership of Janke, Magiera, and colleagues: multiple molecular substructures and functions in the embryonic cerebral cortex (48), cilia and flagella (including spermatogenesis), and cell division. Their research group has independently confirmed and extended our demonstration that polyglutamylation is directly linked to neurodegeneration in *pcd* mice (67) and noted additional pathological changes in spinal cord ventral horn motor neurons and in peripheral nerves (68). Indeed, Janke and his team have presented the major finding of a small cohort of human children (*n* = 13) with an *Nna1* (named CCP1) mutation; these children had severely atrophic cerebella and degenerative changes in spinal cord motor neurons and peripheral nerves, features they also found in *pcd* mice. They have also developed a highly effective purification method for tubulin that will reliably extend analysis of microtubulins and their interactions with other proteins (69).

Two cautionary aspects of this work merit further comment. First, one should emphasize that the molecular seesaw model proposed here should still be considered speculative until future studies adequately distinguish it from other potential neuronal insults that might also result in cell death in this setting. Second, any implications regarding the pathophysiology and phenotypical features of “*pcd*-like” human neurological diseases are not as yet unequivocally clear and should remain open for further investigation. These notable caveats notwithstanding, here we have introduced a molecular seesaw model that might account for the complex and delicate functional and morphological interaction among glutamylation, deglutamylation, and glycylation in mouse models and perhaps in human patients with poorly understood neurodegenerative diseases. Ongoing (70–72) and future studies will establish the ultimate value of this mechanistic hypothesis, and its potential for development of translational applications.

## Methods

### Animals.

We maintained *pcd^Sid^* mutants congenic with the C57BL/6J strain and purchased mice with other *Nna1* mutant alleles (The Jackson Laboratory). Affected progeny were obtained from either heterozygote × heterozygote crosses or homozygous female × heterozygous male crosses. The *pcd* genotyping was analyzed with *Nna1* genome DNA PCR or *Nna1* cDNA sequencing. Adult homozygotes were also identified in segregating litters by their obvious ataxia.

### Abs.

Primary Abs included mouse monoclonal Abs against Calb (calbindin-D-28K; catalog C9848; clone CB-955; MilliporeSigma) for PN and rabbit polyclonal Abs against activated Casp3 (caspase-3; catalog ab4051; Abcam), Calr3 (calreticulin 3; catalog ab254913; Abcam), Nna1 (catalog 4456; Cell Signaling), PolyE (anti-polyglutamylation; catalog GT335; AdipoGen), α-tubulin (catalog AB3201; MilliporeSigma), *Ttll1* (catalog PA5-27285; Invitrogen), and *Ttll3* (catalog PA5-70598; Invitrogen). Secondary Abs were goat anti-mouse (catalog AP142; Sigma-Aldrich) or anti-rabbit IgG (catalog AP132; MilliporeSigma), conjugated with fluorescent dye (FITC or Cy3). Individual Abs were titrated for optimal staining results.

### Cerebellar cell cultures.

P0 WT mouse pups (C57BL/6J) were decapitated. Cerebellum was aseptically and gently separated from the brain stem and meninges were removed in precooled Ca^2+^- and Mg^2+^-free HBSS containing 0.5% glucose. The cerebellum was minced into small pieces, digested in 0.25% trypsin plus 0.05% DNAse I for 3–5 minutes at room temperature, followed by mixing of the tissue in serum-containing medium (Neurobasal medium, 0.5% glucose, 2 mM glutamine, 1% penicillin-streptomycin, and 10% horse serum) (Gibco, Thermo Fisher Scientific). The mix was then centrifuged at 500*g* for 5 minutes at 4°C. The digested tissue was resuspended in a solution of DNAse I and serum-containing medium (1:1), gently titrated into dissociated cells with a fine glass pipette, passed through a 40 μm nylon mesh filter, and recentrifuged at 500*g* for 5 minutes at 4°C. The dissociated cells were resuspended and counted in serum-free medium (Neurobasal medium, 0.5% glucose, 2 mM glutamine, 1% penicillin-streptomycin, 20 μg/mL insulin, 20 μg/mL transferrin, 20 ng/mL sodium selenite, and 2% B27 supplement). Cell suspensions were plated at a density of 3 × 10^6^ cells/mL in poly-L-lysine–coated 16-well Lab-Tek chamber slides (200 μL/well). After maintenance at 37°C with 5% CO_2_ for 6 hours, by which time all cells had attached to the well bottom, and the cell medium was replaced with fresh medium. The cerebellar cells were maintained for 7–28 days in vitro (7–28 DIV), treated with various factors at designated times, and fed once per week by replacing half of the medium. Various Abs were used to assess cerebellar cell morphology, including PN dendritic branchlets.

For PN enrichment, dissociated cerebellar cells from P0 pups were loaded onto cushions of 35% Percoll in EDTA-containing HBSS, and the cells accumulating at the 0%–35% interface were collected. For GN enrichment, dissociated cerebellar cells from P7 pups were put through a 2-step Percoll gradient. The dense cell fraction at the interface between the 35% and 60% Percoll phases was collected. Nonneuronal cells were removed from the above-mentioned enriched preparations by 2 sequential platings on poly-L-lysine–coated Petri dishes. Nonadherent neuronal cells were collected, centrifuged, resuspended in serum-free medium, counted, and plated in poly-L-lysine–coated 16-well Lab-Tek chamber slides. The enriched PNs were cultured at 37°C with 5% CO_2_ for 14 DIV and treated with various factors including specific Abs to assess PN dendritic branchlets and cell survival.

### Lentiviral transduction particle preparation and injection.

The Omicslink expression construct (pReceiver-Lv08 with CMV promoter; GeneCopoeia) was used for packaging, transduction, and stable integration of the *Nna1 or Ttll1* open reading frames into genomic DNA. The pLV-PK-01 kit (GeneCopoeia) contained 2 packaging plasmids (pLV-PK-FIV and pLV-PK-VSG) and a positive lentiviral EGFP expression construct. To generate pseudoviral particles, 293Ta producer cells were grown in 10 cm Petri dishes with DMEM medium containing 15% FBS and antibiotics for 2–3 days (50%–70% confluence) and were washed with serum- and antibiotic-free DMEM before transfection. The washed cells were then transiently cotransfected by adding a transfection complex containing 10 μg of 2 packaging plasmids, 2 μg expression construct, 20 μL Plus Reagent, and 30 μL Lipofectamine Reagent (Invitrogen) in DMEM without serum or antibiotics. After incubation overnight, the transfection complex-containing medium was replaced with fresh DMEM, supplemented with 2% FBS and antibiotics, and incubated for 24–48 hours to produce pseudoviral particles. All pseudovirus-containing medium was collected into 15 mL sterile, capped conical tubes and centrifuged at 2000*g* for 5 minutes at room temperature to pellet cell debris. The supernatant was filtered through a 0.45 μm PVDF filter with low protein binding (Millipore Sigma). The pseudoviral particles collected from 293Ta cell culture media were concentrated by ultracentrifugation at 100,000*g* for 90 minutes at 4°C and titrated with QuickTiter Lentivirus Quantitation Kit (Cell Biolabs). Transductive ability of the titrated pseudoviral particles was also pretested in 293Ta cells before formal use. The titrated pseudoviral particles were adjusted to 5 × 10^6^ TU/mL for transducing cerebellar cells at 2 DIV or 7 DIV and adjusted to 1 × 10^9^ TU/mL for injection into P7 mouse cerebellar cortex.

Mission Lentiviral Transduction Particles (MilliporeSigma) containing the pLKO.1-puro expression construct with U6 promoter were used to transduce Nna1 shRNA or Ttll3 shRNA into cerebellar cells at 7 DIV, which in turn expressed Nna1 siRNA or Ttll3 siRNA to mediate formation of gene-specific RNAi in the transduced cells. Empty viral particles and viral particles with nontargeting shRNA were used as negative controls, and turboGFP particles served as positive controls (MilliporeSigma). Titer of the transduction particles was 5 × 10^6^ TU/mL.

### Histological observation and immunofluorescence staining.

P7-P180 mice were anesthetized deeply with freshly prepared tribromoethanol (Avertin) administered i.p. and the heart was subsequently perfused with fresh 4% paraformaldehyde (PFA; MilliporeSigma). Brain, eyes, and testes were dissected out and kept in the same fixative at 4°C. Cerebellar cryostat sections (10–30 μm thick) were stained with hematoxylin and eosin. Some tissue samples were dehydrated with graded ethanol, embedded in the JB-4 Plus Kit (Ted Pella), sectioned at 1–3 μm, and cresyl violet–stained (Nissl) for cytoplasmic RNA.

PFA-fixed cell cultures and tissue sections were incubated in a blocking solution of 3% goat serum and 0.3% Triton X-100 in PBS at room temperature for 30 minutes and then reacted with specific primary Abs at 4°C for 16–48 hours. Samples were then washed with PBS and stained by secondary Abs at 4°C overnight (ON). After final washing with PBS, the stained samples were sealed with mounting medium containing DAPI (Vector) to stain cell nuclei, and visualized by either confocal microscopy (ZEISS Axiovert 100M) or fluorescence microscopy (Nikon Eclipse E600 or ZEISS Axiophot).

### LCM and cDNA microarray.

To obtain purified PNs and GNs, fresh cerebellar tissues from P20 *pcd^Sid^* and WT littermates were cryosectioned at 7 μm and mounted on special PEN membrane glass slides. The slides were stained with HistoGene frozen section staining kit (Molecular Devices), dehydrated with graded ethanol (75%, 95%, and 100%), made transparent with xylene, and quickly air-dried. An Arcturus LCM instrument (Molecular Devices) was used to isolate individual PN bodies or small clusters of GN cell bodies from cerebellar cortex. A total of approximately 200 PN bodies or approximately 1000 GN bodies were collected in CapSure HS LCM caps and dissolved with dissecting buffer. The PicoPure RNA extraction Kit (Molecular Devices) was used to extract total RNA from the LCM-isolated cells. A BioAnalyzer (Agilent) was used to evaluate the quality and quantity of each RNA sample.

The WT-Ovation Pico RNA Amplification Kit and FL-Ovation cDNA Biotin Module V2 Kit (NuGEN Bioscience) were used in succession to prepare enough qualified cDNA for microarray and qPCR. The amplification process included first-strand cDNA synthesis, second-strand cDNA synthesis, purification of double-stranded cDNA, SPIA amplification, and purification/quantification of amplified SPIA cDNA. The hybridization process included fragmentation of SPIA cDNA, biotin labeling of SPIA cDNA, and GeneChip (mouse genome 430 2.0 array) hybridization with hybridization cocktail (Affymetrix). After washing and staining with an Affymetrix GeneChip array station, the hybridized arrays were scanned with an Affymetrix GeneChip scanner. A confocal laser scanner capable of interrogating individual fluorescence dye-labeled probes and producing separate scanning TIFF images assessed differential gene expression. For image processing and data normalization, dChip and GeneChip operating software (Affymetrix) were used to determine signal intensity (background-subtracted and adjusted for noise) for each transcript. The signal intensity of individual genes and the signal log2 ratio between compared samples were applied to define differential expression. The scanned array images were further analyzed by Microarray Suite software version 5.0 (Affymetrix) to obtain signal intensity, detection call, and detection *P* value. The data were also analyzed with the Affymetrix Data Mining Tool (DMT, version 3.0), NetAFFX, and Microsoft Excel. The confidence bound (≥ 2.0) of the signal log2 ratio between the 2 groups was set as the cutoff criterion.

### Western blot.

Proteins were extracted from cell cultures and tissue samples with a mammalian protein extraction reagent containing a protease inhibitor cocktail and quantified with the BCA Protein Assay Kit (Thermo Fisher Scientific). A total of 50 μg of protein was denatured at 95°C for 3 minutes, separated with 12% SDS-PAGE, and transferred from gel to nitrocellulose membrane (Schleicher & Schuell). The membrane was blocked with 5% fat-free milk at room temperature for 30 minutes and incubated with primary Abs at 4°C ON. After washing, each membrane was incubated with horseradish peroxidase–conjugated secondary Ab for 1 hour at room temperature, washed, and then reacted with enhanced chemiluminescence Western Blotting Detection Reagent (Amersham Bioscience) for 2 minutes. A high-performance autoradiography film (Amersham) was taped onto the Saran-wrapped membrane in a dark room for 2–30 seconds and then developed to visualize the bound Ab. β-actin served as a control protein to eliminate loading variations.

### PCR, RT-PCR, and nucleotide sequencing.

For PCR, genomic DNA was extracted from tail clips. The WT *Nna1* allele (300 bp) was amplified for PCR with forward primer 5′-GGTCAGTTTATTGTGGGTCTC and reverse primer 5′-GTGGGAATACTCTGAGGTCAG. For RT-PCR and sequencing, total RNA was isolated from tissue samples with an RNA Purification Kit (Invitrogen). The quality of purified RNA was detected in agarose gel. Two-step RT-PCR was performed to maximize uniformity of PCR templates for all reactions. Up to 2 μg of total RNA from each sample was reverse-transcribed into the first-strand cDNA in 20-μl volume with oligo(dT) primers and the SuperScript First-Strand Synthesis System (Invitrogen). PCR amplification of the *Nna1* coding sequence was performed with gene-specific oligonucleotide primers and ReddyMix PCR Master Mix (Thermo Fisher Scientific). PCR products were verified with 1% agarose gel and cleaned with ExoSAP-IT (USBiological). The nucleotide sequence of cDNA templates was analyzed at the BIDMC Sequencing Facility with Rhodamine Dye Terminator Ready Reaction Cycle Sequencing Kit (catalog 403044; Thermo Fisher Scientific) with AmpliTaq DNA polymerase FS and specific primers. Samples were electrophoresed, detected, and analyzed on a PE/ABI 377 DNA Sequencer (Applied Biosystems). Sequence data were analyzed with Gene Runner software (http://www.generunner.net/) and the BLAST program.

### Quantitative real-time PCR.

cDNA samples were prepared from LCM-isolated cells and cerebellar cell cultures. SYBR Green I-based qPCR was carried out by DNA Engine Opticon Continuous Fluorescence Detection System (Biocompare). The PCR mixtures contained PCR buffer (10 mM Tris-HCl, 50 mM KCl, 2 mM MgCl_2_, and 0.1% Triton X-100), 250 μM deoxynucleoside triphosphate, 0.5 μM of each specific PCR primer, 0.5x SYBR Green I (Thermo Fisher Scientific), 5% DMSO, 1 unit Taq DNA polymerase (Promega), and 2 μl cDNA in a 25-μl final volume reaction mix. The samples were loaded into wells of Low Profile 96-Well Microplates (Thermo Fisher Scientific). After initial denaturation for 1 minute at 94°C, conditions for cycling were 35 cycles of 30 seconds at 94°C, 30 seconds at 56°C, and 1 minute at 72°C. The fluorescence signal was measured right after incubation for 5 seconds at 79°C following the extension step, which eliminated possible primer dimer formation. At the end of the PCR cycles, a melting curve was generated to identify specificity of the PCR product. For each run, serial dilutions of GAPDH plasmids were used as standards for quantitative measurement of amplified DNA. For normalization of each sample, GAPDH primers served for us to measure the GAPDH cDNA amount. The data are given as ratios of measured gene/GAPDH. PCR products were verified with a 1% agarose gel.

### Protein synthesis.

PN ability to synthesize proteins was measured with Click-iT Plus OPP Protein Synthesis Assay Kit (Thermo Fisher Scientific). The kit contains all the components needed to label and detect the incorporated O-propargyl-puromycin into newly translated proteins on adherent cell samples.

### DNA fragmentation.

DNA fragmentation in PNs was detected with Click-iT TUNEL Assays (Invitrogen). The most widely used in situ test for studying DNA fragmentation or strand breaks is the TUNEL assay, which is based on incorporation of modified dUTPs by terminal deoxynucleotidyl transferase at the 3′-OH ends of fragmented DNA. Each Click-iT TUNEL Kit (Thermo Fisher Scientific) contains all the components necessary to detect DNA fragmentation accurately in adherent cells in culture wells.

### Statistics.

Number and proportion of specifically stained positive or negative cells were counted from cell cultures, or they were quantified from a series of sequentially representative cut tissue sections. Single PNs in cerebellar cell cultures and in the cerebellar cortex were randomly selected and photographed by confocal microscopy with a ×63 oil immersion objective. Quantity I software (Bio-Rad) was used for the quantitative analyses of cDNA and protein bands on gel or film. Comparison between either WT and *pcd* groups or experimental and control groups was performed with 2-tailed Student’s *t* test or 1-way ANOVA. Results were listed as mean ± SD. Differences were rated significant when *P* values were less than 0.05 or very significant when *P* values were less than 0.01.

### Study approval.

Use of animals in the present study was in accordance with NIH-approved institutional guidelines approved by the IACUC at the Beth Israel Deaconess Medical Center and the Harvard Medical School.

## Author contributions

JL, EYS, and RLS designed experiments. JL performed all experiments. JL, EYS, FHFT, RP, WA, and RLS analyzed data. JL and RLS wrote the initial manuscript draft, to which all authors contributed edits. All authors read and agreed with the manuscript content.

## Version 1 10/02/2020

Electronic publication

## Version 2 10/07/2020

Affiliation number 4 was corrected.
